# Enhancing healthcare information security through VAE-driven anomaly detection in EHR access patterns

**DOI:** 10.1038/s41598-026-55024-8

**Published:** 2026-05-26

**Authors:** Touseef Iqbal, Ifrah Raoof, Mohannad Alkanan, Yonis Gulzar

**Affiliations:** 1 UPES Bidholi, School of Computer Science, Dehradun, 248007 India; 2 Department of Computer Science & Engineering, National Institute of Technology, Srinagar, Jammu & Kashmir 190006 India; 3 Department of Management Information Systems, College of Business Administration, King Faisal University, 31982 Al-Ahsa, Saudi Arabia

**Keywords:** Electronic health records (EHRs), Anomaly detection, Variational autoencoder (VAE), Healthcare information security, Data augmentation, Insider threat detection, Privacy-preserving machine learning, Computational biology and bioinformatics, Engineering, Health care, Mathematics and computing

## Abstract

HIPAA breaches and unauthorized access to Electronic Health Records (EHRs) have been growing more likely due to the sudden digitalization of the healthcare sector. High endurance, privacy-based security practices have never been more in demand as hospitals and other medical facilities of this type have clung to the electronic system. The given study considers this problem by suggesting an anomaly detection model that determines the presence of abnormal or suspicious access patterns in EHR systems using Variational Autoencoders (VAEs). One of the main weaknesses of creating an efficient security model in the healthcare sector is that access to real-world information is limited and is constrained by privacy policies. To bridge this challenge, a synthetically enriched EHR access log dataset was generated and realistic features, including departmental affiliations, user roles, frequency of access, and timestamps, were ensured; the artificially generated dataset is, therefore, a close simulation of real-world hospital activities. By observing the access patterns of healthcare professionals that are generally typical in a latent space, the proposed VAE model can signal deviations that can indicate a possible security breach or policy violation without revealing or even relying on actual patient data, thus identifying both large and small-scale aberrations by modelling these latent representations. Evidence of the superiority of VAE-based detection over traditional machine learning algorithms, including Isolation Forest and One-Class Support Vector machines, using some key measures, like accuracy (F1-score: 0.93), lower false-positive rates, and greater sensitivity to noisy data, confirms this assertion. As a result, unsupervised deep generative modelling plus synthetic data generation provides a new, privacy-conserving approach to improving the cybersecurity of medical information systems. Based on the results, the VAE-based anomaly detection can become a trusted means of protecting sensitive healthcare infrastructure against the changing cyber risks.

## Introduction

The terms “security” or “protection” in healthcare can often seem unclear or confusing because they mean different things in different situations. In simple terms, security in healthcare facilities refers to a system of measures designed to protect the property and ensure the safety of everyone who works in or visits the facility^1^. However, what counts as “safe” is not fixed–something safe today might not be safe tomorrow. It’s hard to measure safety because it depends on changing circumstances and personal judgment. The main aim of security is not to completely eliminate all risks but to reduce the chances of harmful incidents and limit the damage if they occur^2.3^. The difficulty lies in detecting the abnormal activities, like unauthorized access to private information, because healthcare data systems are dynamic and distributed^4^. Because insider attacks entail the unusual use of valid credentials, they frequently get past traditional security measures. Therefore, sophisticated analytics that can extract intricate behavioral patterns from access records are necessary to identify such subtle anomalies. Recent developments in deep learning (DL) and machine learning (ML) have created new opportunities for intelligent security monitoring^5^. While supervised machine learning techniques like Random Forests and Support Vector Machines (SVM) have been used for intrusion detection, these models mostly rely on labeled datasets, which are hard to come by or unavailable in the healthcare industry because of privacy regulations^4–6^. Unsupervised techniques have shown potential in detecting abnormalities without the need for explicit labels, especially deep generative models. The ability to learn compressed latent representations of normal data distributions and to reconstruct input patterns with minimal loss makes Variational Autoencoders (VAEs) unique among them^7^. In EHR systems, VAEs are a good option for simulating typical user access behaviors since deviations in reconstruction error are useful signs of anomalies. Despite these advancements, there are still a number of gaps in the use of deep generative models for the security of healthcare data. Most recent research focuses on network intrusion detection rather than user behavior modeling in EHR environments^8^. Furthermore, creating strong models that can generalize across real-world settings is made difficult by privacy issues and restricted data availability. Both methodological innovation and synthetic data augmentation techniques that replicate real EHR access patterns while maintaining confidentiality are needed to address these issues.

**Fig. 1:**
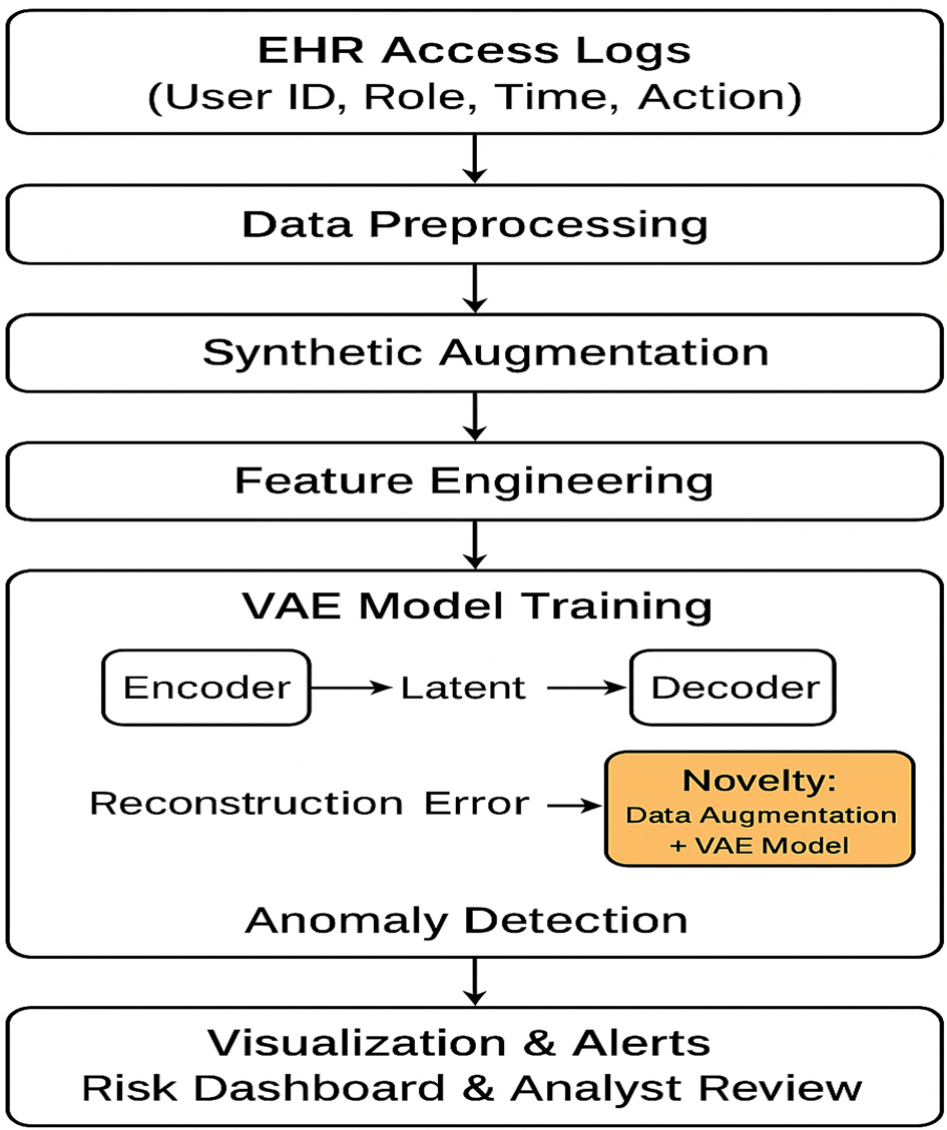
VAE-based anomaly detection framework for secure EHR access.

A VAE-based anomaly detection framework is proposed in this study to detect anomalous access behaviors in healthcare data systems. Using realistic elements, including user roles, departments, timestamps, access frequency, and data sensitivity levels, a synthetically enhanced EHR access log dataset was produced in order to address the issue of data scarcity. The proposed model detects deviations that may indicate potential insider threats or data breaches by analyzing the latent space representation of common access behaviors. Because the VAE architecture may independently capture nonlinear behavioral dependencies, it is flexible and privacy-preserving compared to traditional rule-based or threshold-driven systems. This research is innovative because it develops a scalable and privacy-aware anomaly detection system for the medical industry by combining data augmentation techniques with unsupervised generative modeling. By providing an interpretable probabilistic mechanism for anomaly scoring, VAEs lower false-positive rates in this scenario compared to more conventional unsupervised techniques like Isolation Forest and One-Class SVM. Additionally, visualization elements that bridge the gap between deep learning interpretability and cybersecurity decision-making, including reconstruction error heatmaps and latent space clustering, make it simple for security analysts to spot odd patterns. The top-down structure of this study is shown in Fig. 1.

The following summarizes this study’s primary contribution: In addition to detecting generic anomalies, the proposed methodology aims to identify specific security vulnerabilities to healthcare systems and HIPAA compliance. These include unauthorized cross-departmental access, excessive retrieval of patient records, which may imply data exfiltration, irregular access outside of working hours, and insiders abusing genuine credentials. By modeling regular access behavior and detecting deviations, the system allows for early detection of unusual activity that may indicate policy violations or security breaches in EHR systems.

- A system for simulating EHR access logs while protecting privacy that produces realistic synthetic data for model validation and training.
- A VAE-based anomaly detection system that doesn’t need labeled data to learn latent access behavior patterns.
- In-depth performance analysis and visualization that highlights the suggested model’s detection accuracy, efficiency, and interpretability by comparing it with baseline techniques.

The rest of this paper is organized as follows: The related work on anomaly detection and healthcare data security is reviewed in section “Related work”. The methodology, along with information on model design, dataset creation, and implementation specifics, is described in section “Proposed methodology”. The experimental setup and evaluation metrics are covered in section “Experimental setup”. The data and analysis are presented in detail in section “Proposed model architecture”. The limits and future work are covered in section “Results and analysis”, and the paper is concluded in section “Limitations and future work”.

## Related work

A developing field of study that combines information security, anomaly detection, and healthcare informatics is the detection of harmful or unauthorized access to Electronic Health Records (EHRs). In the healthcare area, insider threats and subtle misuse of legitimate credentials are often difficult to detect with traditional security controls like firewalls, encryption, and role-based access control (RBAC). These threats usually appear as unusual user behavior rather than obvious network signatures. Recent surveys of insider-threat detection and security monitoring underscore this shift toward behavior-centric analytics and the limitations of perimeter defenses^9,10^. Machine learning (ML) and deep learning (DL) have been widely adopted for security monitoring over the last decade. Early and influential work applying deep architectures to intrusion detection illustrated the value of representation learning for complex, high-dimensional measure such as network flows and system logs^5^. Many of these systems, however, focused on network-level anomalies rather than user-level behavior in application logs or EHR access audits. This distinction matters because network anomalies (e.g., DDoS, port scanning) and user behavior anomalies (e.g., unusual patient-record reads) have different statistical signatures and require different modeling choices.

Unsupervised methods for anomaly detection have therefore attracted particular attention. Autoencoders and their probabilistic variants–notably Variational Autoencoders (VAEs)–are appealing because they learn compact latent representations of normal behavior and flag deviations based on reconstruction error or latent-space improbability. The seminal VAE formulation established the probabilistic basis for amortized variational inference and reparameterization, enabling scalable training of reconstruction-based anomaly detectors^11^. More recent work has adapted VAEs to time series and multivariate measures (e.g., LSTM/GRU encoders/decoders, frequency-aware conditional VAEs) to better capture temporal patterns that are central to user behavior modeling^12^. In the context of healthcare specifically, recent transformer-based generative models (e.g., TransformEHR, Foresight) have demonstrated that powerful sequence models can learn rich patient-timeline representations from longitudinal EHR data^13^. While most such work focuses on prediction of clinical outcomes, pretraining and generative capabilities illustrate an alternate path: generative sequence models can be adapted for behavior modeling and anomaly detection when suitably retrained on access-log sequences rather than clinical measurements. Another important strand in the literature is synthetic data generation and privacy-preserving augmentation. Privacy barriers often prevent sharing or centralization of audit logs for model training. Recent advances in diffusion models and hybrid autoencoder-diffusion architectures have produced realistic synthetic EHR time series that preserve statistics needed for downstream model training while minimizing disclosure risk^14^. Synthetic augmentation is therefore a pragmatic way to bootstrap anomaly detectors and to simulate attack scenarios for evaluation without exposing Protected Health Information (PHI). There are also multiple efforts to improve robustness, interpretability, and deployment readiness. Frequency-aware VAEs and ensembles (combining reconstruction scores with density estimation or traditional anomaly detectors like Isolation Forest) address shortcomings in VAE reconstructions for long-periodic and heterogeneous signals. Explainability methods (like gradients or feature-based attributions) help security analysts understand alerts better by showing which data fields had the biggest impact on why a system flagged something as unusual^9^. Finally, privacy-focused training methods (like DP-SGD and federated learning) are becoming practical ways for different organizations to train models together without having to share or combine their sensitive audit data in one place^15^. Recent advances in anomaly detection have focused on deep learning and sequence-based models, such as recurrent neural networks and transformer-based architectures, that can describe temporal connections in sequential data. These methods have proven effective in collecting complicated behavioral patterns in time series and access logs.

### Gaps and opportunities

Even with these improvements, there are still challenges in applying these methods to monitoring EHR (Electronic Health Record) access. Most past studies focus on network intrusion detection or clinical prediction, not on detailed modeling of user access behavior. Testing on real audit logs is also rare, and very few studies combine synthetic EHR access data with unsupervised VAE-based detection methods. Furthermore, there is room for new research that links cutting-edge techniques with practical applications because practical concerns, including setting role-based baselines, employing dynamic thresholds to reduce false alarms, and monitoring model drift, are not thoroughly examined. A summary of the techniques for identifying irregularities and insider threats in the healthcare industry and adjacent domains is provided in Table 1. It displays each method’s primary strengths, significant reference studies, and areas of application, including whether supervised or unsupervised learning is used. All things considered, it encapsulates the ways in which conventional and contemporary methods vary with regard to their interpretability, simplicity, and ability to accurately simulate user access behavior in healthcare environments.

**Table 1:** Summary of existing approaches for anomaly and insider threat detection in healthcare and related domains.

Technique Category	Application Domain	Representative Models / Methods	Supervised?	Strengths	Example References
Rule-based / Heuristic	Network, EHR audit	Thresholds (off-hours, access volume)	No	Simple, interpretable, low computational cost	–
Statistical (PCA, z-score)	Network, logs	PCA reconstruction error, z-score deviation	No	Fast, interpretable anomaly scoring	^16^
Traditional ML (SVM, RF)	Network, host logs	Support Vector Machines, Random Forest	Yes (labeled data)	Effective with labeled datasets, generalizable	^17^
Autoencoders (AE, DAE)	Network, session logs	Dense AE, Stacked AE	Unsupervised	Learns non-linear feature representations	^18^
Sequence AE (LSTM-AE)	Sequential user sessions	LSTM encoder-decoder AE	Unsupervised	Captures temporal access behavior patterns	^19^
Variational Autoencoders (VAE)	Time series, EHR sessions	VAE, *β*-VAE, conditional VAE	Unsupervised	Probabilistic latent modeling, robust reconstruction	^20^
Frequency-aware / Conditional VAE	Time series, event logs	FCVAE, VRNN	Unsupervised	Handles multi-frequency temporal patterns	^12^
Transformer-based Models	EHR timelines, activity logs	TransformEHR, Foresight	Self-/Unsupervised	Captures long-term dependencies, contextual learning	^13^
Diffusion / Hybrid AE-Diffusion	Synthetic EHR data generation	DDPM, ScoEHR	–	Generates realistic privacy-preserving synthetic data	^21^
One-Class / Ensemble Models	Any log data	Isolation Forest, One-Class SVM, AE+SHAP hybrids	Semi-/Unsupervised	Robust, interpretable, complementary detection layer	^9^

## Proposed methodology

The Differentially Private Secure Variational Autoencoder (DP-S-VAE) framework is presented in this section as a means of guaranteeing the privacy, anonymity, and safe reconstruction of medical data. To transform sensitive patient data into anonymized latent representations appropriate for secure data analysis, the technique integrates probabilistic representation learning, information-theoretic security, and differential privacy.

### Problem definition and system architecture

Let the healthcare dataset be represented as:

1 $$\begin{aligned} \mathscr {D} = \left\{ x^{(i)} \in \mathbb {R}^d \;\bigg |\; i = 1, 2, \dots , N \right\} \end{aligned}$$

This is how the notation is shown: $x^{(i)}$ indicates the *i-th* patient record or medical sample (e.g., 3. lab results, electronic health record (EHR) attributes), and *d*: indicates the dimensionality of the data instance (i.e., E. number of features). *N*: total records in the dataset. The whole patient sample from the medical database is shown as $\mathscr {D}$. Three primary neural components make up the proposed model: The **Encoder Network** $q_{\phi }(z|x)$ encodes the input *x* into a latent probabilistic space *z*, producing distribution parameters $\mu _{\phi }(x)$ and $\Sigma _{\phi }(x)$. The **Security Layer** *S(· )* adds controlled Gaussian noise to the latent variable *z* in order to protect privacy and ensure differential privacy compliance. Finally, the **Decoder Network** $p_{\theta }(x|z')$ rebuilds an anonymized version $\hat{x}$ using the perturbed latent variable *z'*.

### Variational autoencoder (VAE) formulation

This architecture promotes privacy by transforming raw input data into abstract latent representations that do not contain immediately identifiable information. The random nature of the encoding makes it even more difficult to attribute specific access events to individual users or patients. The generative mechanism assumed by the Variational Autoencoder (VAE) is described as follows:

2 $$\begin{aligned} p_{\theta }(x, z) = p_{\theta }(x|z)\,p(z) \end{aligned}$$

Several key components are outlined in the model. The joint distribution $p_{\theta }(x, z)$ represents the correlation between the latent variable *z* and the input data *x*, parameterized by the model weights *θ*. The distribution across latent variables is described by the prior *p*(*z*), which is usually taken to be a conventional normal distribution. This is $\mathscr {N}(0, I)$. The decoder, or likelihood $p_{\theta }(x|z)$, models the reconstruction of *x* from the latent variable *z*. Lastly, the latent (hidden) form of the medical data is indicated by $z \in \mathbb {R}^k$, where *k ≪ d*. The encoder approximates the true posterior $p_{\theta }(z|x)$ by a variational distribution:

3 $$\begin{aligned} q_{\phi }(z|x) = \mathscr {N}\big (z; \mu _{\phi }(x), \Sigma _{\phi }(x)\big ) \end{aligned}$$

The neural network weights *ϕ* parameterize the inference distribution $q_{\phi }(z|x)$, which is defined by the encoder. Together, the mean vector $\mu _{\phi }(x)$ and the diagonal covariance matrix $\Sigma _{\phi }(x)$ that the encoder produces for a given input *x* guarantee a stochastic encoding of the input. The decoder reconstructs the input as:

4 $$\begin{aligned} p_{\theta }(x|z) = \mathscr {N}\big (x; f_{\theta }(z), \sigma _x^2 I\big ) \end{aligned}$$

The VAE operates in three steps. First, the encoder network converts the input feature vector into a latent representation with mean and variance, capturing the underlying structure of typical access behavior. Second, a latent vector is drawn from this distribution using the reparameterization approach, which introduces controlled stochasticity while preserving differentiability. Third, the decoder reconstructs the input from the sampled latent vector, attempting to match the original access pattern as closely as feasible. The neural network implements a non-linear transformation $f_{\theta }(z)$, which is applied by the decoder. *I* represents the identity matrix of the relevant dimension, and the reconstruction noise has variance $\sigma _x^2$, which regulates the uncertainty in reconstructing the input.

### latent sampling via reparameterization

To allow backpropagation through stochastic sampling, the *reparameterization trick* is used:

5 $$\begin{aligned} z = \mu _{\phi }(x) + \Sigma _{\phi }^{1/2}(x) \odot \epsilon , \quad \epsilon \sim \mathscr {N}(0, I) \end{aligned}$$

The model employs the element-wise multiplication operator *⊙* and adds auxiliary random noise *ε* sampled from a unit normal distribution $\mathscr {N}(0, I)$. Gradient-based optimization via the stochastic latent variable is made possible by this process, which guarantees that *z* stays differentiable with respect to the encoder parameters *ϕ*.

### Evidence lower bound (ELBO) objective

The learning objective of the VAE is to maximize the *Evidence Lower Bound (ELBO)*:

6 $$\begin{aligned} \mathscr {L}(\theta , \phi ; x) = \mathbb {E}_{q_{\phi }(z|x)} \big [ \log p_{\theta }(x|z) \big ] - D_{\text {KL}} \big ( q_{\phi }(z|x) \, \Vert \, p(z) \big ) \end{aligned}$$

To regularize the latent distribution to be near the prior *p*(*z*), the model employs $D_{\text {KL}}(\cdot \, \Vert \, \cdot )$, the Kullback-Leibler divergence, and $\mathbb {E}_{q_{\phi }(z|x)}[\cdot ]$, the expectation under the approximate posterior distribution $q_{\phi }(z|x)$. While the second term guarantees that latent variables stay near the previous, preventing memorization of identifying patient information, the first term promotes accurate reconstruction of healthcare data. Using the *β*-VAE formulation, privacy control is further strengthened.

7 $$\begin{aligned} \mathscr {L}_{\text {total}} = \mathbb {E}_{q_{\phi }(z|x)} \big [ \log p_{\theta }(x|z) \big ] - \beta \, D_{\text {KL}} \big ( q_{\phi }(z|x) \, \Vert \, p(z) \big ) \end{aligned}$$

where information leakage from the latent space is decreased and stronger latent regularization is enforced by the coefficient *β > 1*.

### Latent security and differential privacy

To prevent reconstruction from intercepted latent variables, a Security Layer adds Gaussian perturbation:

8 $$\begin{aligned} z' = S(z) = z + \eta , \quad \eta \sim \mathscr {N}(0, \sigma _s^2 I) \end{aligned}$$

the notation is like the latent vector *z'* is transmitted or stored instead of *z*. Random Gaussian noise *η* is added for privacy protection, and the variance $\sigma _s^2$ controls the noise magnitude, where higher $\sigma _s^2$ provides stronger privacy but reduces reconstruction utility. The proposed framework uses privacy-preserving procedures like latent space transformation and stochastic sampling, but it lacks a formal differential privacy guarantee (e.g., *ε* differential privacy). As a result, privacy claims rely on practical observations rather than rigorous theoretical justifications.

#### Differential privacy guarantee

A mechanism *M* satisfies $(\epsilon , \delta )$-*Differential Privacy (DP)* if:

9 $$\begin{aligned} \Pr [M(D) \in S] \le e^{\epsilon } \Pr [M(D') \in S] + \delta \end{aligned}$$

for any two neighboring datasets *D, D'* differing in a single patient record. In our context, the encoder-security combination acts as the mechanism *M*. For Gaussian noise with sensitivity *Δ f*, the DP constraint is satisfied if:

10 $$\begin{aligned} \sigma _s \ge \frac{\Delta f \sqrt{2 \ln (1.25/\delta )}}{\epsilon } \end{aligned}$$

The privacy parameters include *ε*, the privacy budget where smaller values indicate stronger privacy, and *δ*, the failure probability representing the chance that the privacy guarantee does not hold. *Δ f* denotes the $L_2$ sensitivity of the encoder function, which is the maximum change in the latent output when a single patient record is modified. Gaussian noise with standard deviation $\sigma _s$ is added to the latent variable to ensure differential privacy.

### Information-theoretic privacy bound

The information leakage between input data *X* and the privatized latent code *Z'* is quantified by *Mutual Information (MI)*:

11 $$\begin{aligned} I(X; Z') = \mathbb {E}_{x, z'} \left[ \log \frac{p(x, z')}{p(x)p(z')} \right] \end{aligned}$$

For Gaussian perturbation *z' = z + η*, the upper bound of MI is:

12 $$\begin{aligned} I(X; Z') \le \frac{1}{2} \log \left| I + \frac{\Sigma _z}{\sigma _s^2 I} \right| \end{aligned}$$

The latent variables have a covariance matrix $\Sigma _z$, and *I* denotes the identity matrix of dimension *k*, corresponding to the latent dimension. In order to protect privacy, the latent variables are subjected to Gaussian noise with variance $\sigma _s^2$. Based on this relationship, increasing $\sigma _s^2$ noise variance enhances privacy by exponentially decreasing the information flow between *X* and *Z'*.

### Secure objective function

Integrating the privacy constraint, the Secure VAE (S-VAE) objective becomes:

13 $$\begin{aligned} \mathscr {L}_{\text {secure}}(\theta , \phi ; x) = \mathbb {E}_{q_{\phi }(z|x)} \big [ \log p_{\theta }(x|z + \eta ) \big ] - \beta \, D_{\text {KL}} \big ( q_{\phi }(z|x) \, \Vert \, p(z) \big ) \end{aligned}$$

subject to the differential privacy constraint:

14 $$\begin{aligned} \sigma _s \ge \frac{\Delta f \sqrt{2 \ln (1.25/\delta )}}{\epsilon } \end{aligned}$$

Even if latent codes are revealed, this method guarantees that the parameters $(\epsilon , \delta , \sigma _s)$ mathematically restrict the quantity of recoverable patient data.

### Network design

In Table 2, the design of the DP-S-VAE model is provided. Each patient record’s mean ($\mu _\phi$) and log-variance ($\sigma ^2$) are produced by the encoder with two dense layers of 512 and 256 units, respectively, following a ReLU activation. To ensure privacy, the security layer creates a safe latent variable (*z'*) by injecting additive Gaussian noise. Lastly, the decoder creates an anonymized reconstruction ($\hat{x}$) of the input data by mirroring the encoder with dense layers of 256 and 512 units, utilizing ReLU and Sigmoid activations.

**Table 2:** Architecture of the DP-S-VAE model.

Component	Layers	Activation	Output Description
Encoder	Dense(512) *→* ReLU *→* Dense(256) *→* $[\mu , \log (\sigma ^2)]$	ReLU	Produces mean ($\mu _\phi$) and log-variance ($\sigma ^2$) for each patient record
Security Layer	Additive Gaussian Noise	–	Produces secure latent variable (*z'*)
Decoder	Dense(256) *→* ReLU *→* Dense(512) *→* Sigmoid	Sigmoid	Generates anonymized reconstruction ($\hat{x}$)

### Loss functions

The training of the DP-S-VAE minimizes the following loss components:

15 $$\begin{aligned} \mathscr {L}_{\text {total}} = \underbrace{\Vert x - \hat{x} \Vert _2^2}_{\mathscr {L}_{\text {reconstruction}}} + \beta \, \underbrace{\frac{1}{2} \sum _{j=1}^{k} \left( \mu _j^2 + \sigma _j^2 - \log (\sigma _j^2) - 1 \right) }_{\mathscr {L}_{\text {KL}}} \end{aligned}$$

The rebuilt sample of patient data is indicated by $\hat{x}$ in this formulation, while the mean and standard deviation of the $j^\text {th}$ latent dimension are represented by $\mu _j$ and $\sigma _j$. *k* represents the total number of latent dimensions. Stochastic gradient descent is used to minimize $\mathscr {L}_{\text {total}}$ using the *Adam* optimizer with a learning rate *α*.

### Hyperparameter optimization

Five-fold cross-validation and a systematic grid search were used to optimize the model’s hyperparameters. Table 3 provides a summary of the search space and ideal values. With early stopping based on validation loss (patience=20 epochs), training convergence was usually reached in 150–200 epochs. The default momentum settings ($\beta _1=0.9$, $\beta _2=0.999$) were used while using the Adam optimizer.

**Table 3:** Hyperparameter optimization results.

Parameter	Search Space	Optimal Value	Rationale
Latent dimension (*k*)	$\{16, 32, 64, 128\}$	32	Balances reconstruction fidelity and computational efficiency
*β* parameter	$\{0.1, 0.5, 1.0, 2.0, 5.0\}$	2.0	Stronger privacy regularization without significant utility loss
Learning rate (*α*)	$\{0.1, 0.01, 0.001, 0.0001\}$	0.001	Stable convergence without oscillation
Batch size	$\{32, 64, 128, 256\}$	64	Optimal trade-off between gradient estimation and memory usage
Encoder layers	$\{[512,256], [256,128], [512,256,128]\}$	[512, 256]	Sufficient capacity without overfitting

The DP-S-VAE model’s hyperparameter optimization findings are compiled in Table 3. Each choice is explained along with the primary parameters, their search spaces, and the ideal values that were selected. By balancing reconstruction accuracy, privacy protection, and computational efficiency, these parameters were chosen to guarantee that the model effectively anonymizes patient data while maintaining high utility for additional scientific investigation.

### Algorithm: differentially private secure VAE (DP-S-VAE)

**Algorithm 1:**
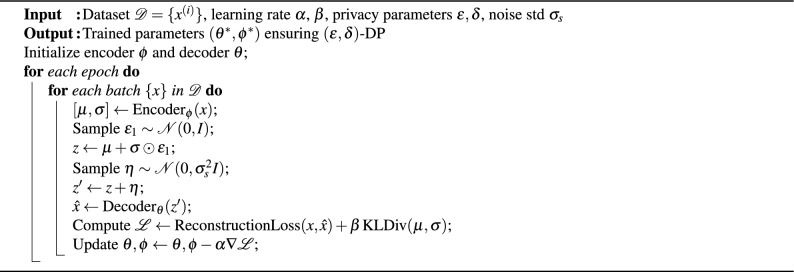
DP-S-VAE Training Procedure

The algorithm "Algorithm: differentially private secure VAE (DP-S-VAE)" describes the training procedure for the Differentially Private Secure Variational Autoencoder (DP-S-VAE). The input includes the patient dataset $\mathscr {D} = \{x^{(i)}\}$, learning rate *α*, KL weight *β*, differential privacy parameters $\epsilon , \delta$, and Gaussian noise standard deviation $\sigma _s$. The encoder parameters *ϕ* and decoder parameters *θ* are first initialized. For each batch of input data *x*, the encoder maps *x* to the latent space, producing mean *μ* and standard deviation *σ*. A random noise $\epsilon _1 \sim \mathscr {N}(0, I)$ is sampled and combined with *μ* and *σ* to generate the stochastic latent vector $z = \mu + \sigma \odot \epsilon _1$. Additional Gaussian noise $\eta \sim \mathscr {N}(0, \sigma _s^2 I)$ is added in the security layer, producing the privatized latent variable *z'*. To reconstruct the input, the decoder uses the formula $\hat{x} = \text {Decoder}_\theta (z')$. Total loss $\mathscr {L}$ is calculated as the sum of the KL divergence term weighted by *β*, which regularizes the latent space, and reconstruction loss, which quantifies the difference between $\hat{x}$ and *x*. In order to guarantee proper reconstruction and privacy compliance, the encoder and decoder parameters $(\phi , \theta )$ are finally modified by gradient descent in order to decrease $\mathscr {L}$. A trained DP-S-VAE model that provides differential privacy guarantees and anonymizes patient data is created by repeating this procedure across all batches and epochs.

## Experimental setup

### Evaluation metrics

The performance of the proposed DP-S-VAE model is assessed using a combination of reconstruction, anomaly detection, and privacy-preserving metrics. To fully measure the model’s efficacy, these metrics are divided into three areas. The privacy and utility of the DP-S-VAE model are evaluated using the following metrics. The reconstruction error, a measure of the data’s utility, is explained as follows.

16 $$\begin{aligned} E_{\text {rec}} = \frac{1}{N} \sum _{i=1}^{N} \Vert x^{(i)} - \hat{x}^{(i)} \Vert _2^2. \end{aligned}$$

The quantification of privacy leakage is done using mutual information between the input and the privatized latent representation.

17 $$\begin{aligned} I(X; Z') = H(X) - H(X|Z'). \end{aligned}$$

The differential privacy constraint enforces the Gaussian noise standard deviation.

18 $$\begin{aligned} \sigma _s \ge \frac{\Delta f \sqrt{2 \ln (1.25/\delta )}}{\epsilon }. \end{aligned}$$

Finally, the performance of anomaly detection is measured using precision, recall, and F1-score.

19 $$\begin{aligned} \text {Precision}&= \frac{TP}{TP + FP},&\text {Recall}&= \frac{TP}{TP + FN},&\text {F1-Score}&= 2 \cdot \frac{\text {Precision} \cdot \text {Recall}}{\text {Precision} + \text {Recall}}. \end{aligned}$$

### Dataset description

A fully synthetic EHR access log dataset was created in order to assess the suggested Secure Variational Autoencoder (S-VAE) architecture in a healthcare security setting. 2,000 log entries representing 50 fictitious users are included in this dataset, which replicates operational settings in the healthcare industry. The initial dataset size is constrained; however, it is gradually enlarged using controlled data augmentation approaches to promote diversity while keeping the underlying statistical features. This technique aims to replicate a wider range of access scenarios without adding arbitrary patterns. A single access event is represented by each log entry, which also has the following characteristics: The dataset contains several fields that describe each access event. In 2025, the date and access time are randomly distributed and recorded by *Timestamp*. Every *User ID* is a distinct identification number between U001 and U050, and *Role* indicates the user’s role inside the hospital, such as administrator, nurse, or doctor. The department linked to the event, such as cardiology, oncology, neurology, pediatrics, and radiology, is indicated in the *Department* field. The number of patient records accessed is indicated by *Records Accessed*, while the type of access (View, Download, or Modify) is described by *Action*. Whether the access was encrypted is indicated by a *Encrypted Flag* (all events set to 1), and 5% of events are marked as anomalous by a binary indicator called *Anomaly*, which is produced by assigning unusual departments or abnormally high quantities of records accessed. The dataset was designed to be fully replicable and privacy-preserving so that studies on latent-space encoding, differential privacy, and anomaly detection could be carried out without using real patient data. To achieve realism, the dataset generation process is governed by domain-specific constraints that mimic real-world hospital procedures. User roles are assigned to normal departments (for example, doctors with clinical departments and administrators with system-level access), and access patterns reflect realistic distributions in terms of frequency, time, and action kinds. Normal behavior is designed to reflect everyday processes, such as moderate record access during working hours, while ensuring consistency across user roles and permissible actions. These design decisions ensure that the resulting data closely resembles real-world EHR access logs. Random variability was included to reflect real access patterns, even though anomalies provide a controlled method to evaluate the model’s ability to detect strange or perhaps illegal access events.

**Table 4:** Sample entries from the synthetic EHR access log dataset.

Timestamp	User ID	Role	Department	Action	Records Accessed	Encrypted Flag	Anomaly
2025-03-15 09:12	U001	Doctor	Cardiology	View	3	1	0
2025-07-22 14:35	U024	Nurse	Oncology	Download	2	1	0
2025-01-05 18:47	U013	Admin	Radiology	Modify	1	1	0
2025-11-10 07:23	U007	Doctor	Neurology	View	8	1	1
2025-06-02 12:50	U032	Nurse	Pediatrics	Download	4	1	0

The synthetic EHR access log dataset is shown in Table 4. A single hospital user access event is represented by each row. The columns contain the following information: the event timestamp, user ID, role, department, action type, number of records accessed, encryption status, and a binary anomaly indicator, which is:

- *Normal Access Events:* When a user accesses records inside their department and the quantity of records accessed falls within a regular range, this is usual user behavior. Rows with Anomaly = 0 in the table represent typical occurrences. Common access events, for instance, are displayed in the first, second, third, and fifth rows.
- *Anomalous Access Events:* Five percent of the dataset consists of these events, which are purposefully included to mimic unusual or possibly illegal access. Unusually high record access counts or access to a department unrelated to the user’s function are two examples of these. In the table, anomalies are indicated by rows with Anomaly = 1. The image’s fourth row, for example, shows a rare occurrence.

By making this distinction, we are able to assess the Secure VAE’s reconstruction performance, its value in hiding sensitive data, and its capacity to identify irregularities in medical access records. The dataset includes a sample of typical user jobs seen in healthcare systems, such as doctors, nurses, administrative staff, and technical professionals. These positions are related with departmental connections including cardiology, radiology, emergency services, and laboratory departments. During data generation, accurate role-department mappings are used to mirror conventional hospital procedures. For example, clinical professions such as doctors and nurses are primarily associated with patient-care departments, but administrative functions may have broader or system-wide access. This structured mapping enables the model to learn consistent access patterns under normal conditions. Anomalous events are caused by purposely disturbing these linkages, such as allocating users to unrelated departments or mimicking activities that are not appropriate for their roles. This enables the dataset to capture both normal and pathological behavioral patterns in an accurate manner. The dataset is synthetically generated, but it is built using domain-informed rules that mimic real-world healthcare operations, such as role-based access patterns, departmental affiliations, and temporal behavior. This guarantees that the provided data reflects meaningful access patterns rather than arbitrary distributions.

### Data augmentation

While maintaining realistic data patterns, we used a thorough data augmentation technique to increase our initial dataset of 2,000 records to 10,500 records in order to address possible class imbalance and strengthen the robustness of our anomaly detection method.

#### Augmentation methodology

To generate imaginative yet realistic EHR access patterns, our augmentation strategy used a variety of techniques: *Temporal Variation:*Timestamp jittering with a maximum variance of *± 10* minutes is used to create realistic timing fluctuations while preserving temporal coherence. *Access Pattern Variations:* Record count jittering is used with fluctuations of *± 3* records to simulate realistic changes in user access behavior while maintaining a minimum threshold of 1 record to ensure data quality. *Contextual Anomalies:*There is a 5 percent chance that applying department reassignment will result in minor contextual changes that indicate questionable behavior.

#### Anomaly enrichment

We created extra artificial anomalies to guarantee accurate representation of anomalous patterns, which made up 5% of the final dataset: *Enhanced Features of Anomalies:* Explicit anomaly labeling to support supervised learning, significant temporal deviations of *± 180* minutes to capture unusual access times, extreme record access counts with 10–50 additional records beyond normal ranges, and role and department reassignment to simulate privilege misuse are several examples.

#### Dataset composition

The following features shown in Table 5 were produced by the augmentation process for the dataset:

**Table 5:** Dataset composition after data augmentation.

Dataset	Records	Anomaly rate	Description
Original	2,000	5%	Initial synthetic data
Augmented Normal	10,000	–	Jittered normal access patterns
Additional Anomalies	500	100%	Enhanced anomalous patterns
Final Combined	10,500	9.5%	Shuffled training dataset

#### Quality assurance

Significant feature correlations present in the original data were maintained during the augmentation process, which also ensured temporal consistency within plausible operating hours, maintained realistic data distributions across roles, departments, and actions, and provided a balanced representation of both common and uncommon access patterns. This all-encompassing approach guarantees that the statistical characteristics of authentic EHR access patterns are preserved while training the anomaly detection algorithms on a variety of realistic scenarios.

### Dataset preprocessing

Before training the Secure VAE, the dataset was preprocessed to ensure numerical compatibility, model stability, and reproducibility as shown in Table 6.

**Table 6:** Feature types and preprocessing methods for the synthetic EHR access log dataset.

Feature type	Column(s)	Preprocessing method	Significance in secure VAE
Categorical	role, department, action, user_id	One-hot encoding	Converts categories to numerical vectors; preserves distinct categories without ordinal bias
Continuous	records_accessed	Min-max scaling [0,1]	Stabilizes neural network training and prevents domination of latent space by large values
Timestamp	timestamp	Extract hour_of_day, day_of_week	Captures temporal access patterns while obfuscating exact times
Binary	encrypted_flag, anomaly	Keep as 0/1	Maintains original flags; anomaly can be used for evaluation

The selected features capture multidimensional user behavior in EHR systems. Role and department features convey organizational structure, whereas action type and records accessed indicate user activity intensity. Temporal variables such as time of day and day of week record routine access patterns. By incorporating these characteristics, the model learns normal behavioral profiles such as role-consistent access, usual record usage, and regular access scheduling. The preprocessing procedure included:

1. *Categorical encoding:* One-hot encoding of roles, departments, actions, and user IDs.
2. *Continuous scaling:* Min-max normalization of ‘records accessed‘ to the range [0,1].
3. *Temporal feature extraction:* Deriving ‘hour of day‘ and ‘day of week‘ from the timestamp.
4. *Data splitting:* 70% training, 15% validation, 15% test with a fixed random seed for reproducibility.
5. *Noise handling:* No differential privacy noise was added at this stage; noise injection occurs in the latent space via the Security Layer.

For the VAE to train steadily and effectively, preprocessing makes sure that all features are numerical and normalized. It prevents any characteristic from unduly affecting the latent representations while maintaining temporal and categorical patterns in the access logs. Furthermore, reproducibility and consistent application of latent-space privacy methods are made possible by deterministic preprocessing, which permits a thorough assessment of the trade-off between privacy and usefulness as well as the model’s capacity to reconstruct and obscure critical access patterns.

### Implementation details

Python 3.9 was used to implement the suggested framework with PyTorch 2.0. Matplotlib was used for visualization, SHAP for interpretability, and Scikit-learn for preprocessing. Training took place on 10GB VRAM-equipped NVIDIA RTX 3080 GPUs.

The code architecture follows modular design principles:

- data_loader.py: Synthetic dataset generation and preprocessing
- models.py: VAE architecture and loss functions
- trainer.py: Training loops and validation
- evaluator.py: Performance metrics and visualization
- explainer.py: SHAP analysis and interpretability

The complete code repository, along with documentation and reproduction instructions, will be made publicly available upon publication to facilitate research reproducibility.

## Proposed model architecture

**Fig. 2:**
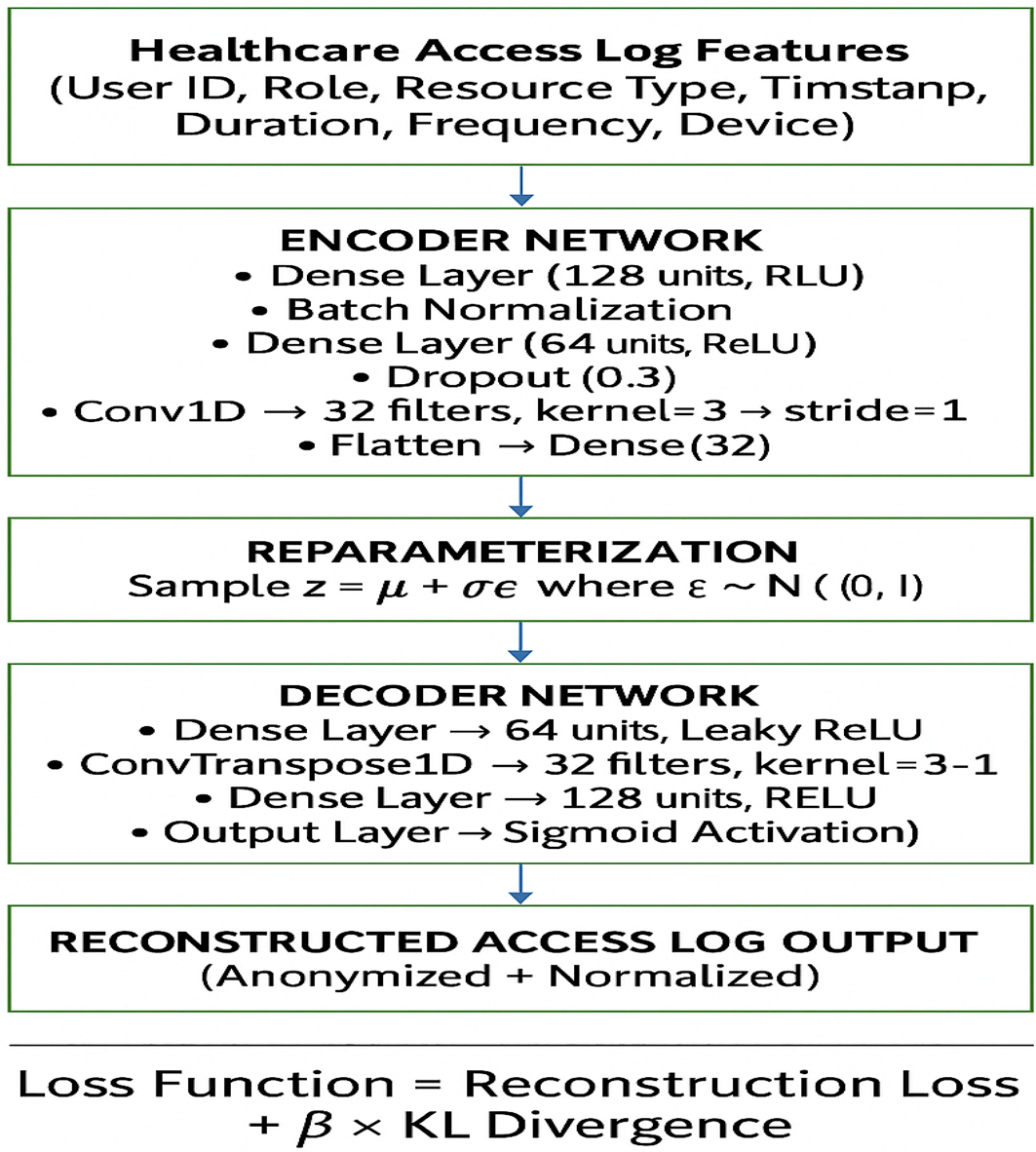
Variational Autoencoder (VAE) architecture for privacy-preserving modeling of healthcare access logs.

*Model architecture.* In order to model healthcare access records, the suggested Variational Autoencoder (VAE) learns a compact and privacy-preserving latent representation, as shown in figure  2. Two functions, $\boldsymbol{\mu }_\phi (\mathbf{x})$ and $\boldsymbol{\sigma }_\phi (\mathbf{x})$, which correspond to the mean and standard deviation of the latent distribution, are parameterized by the encoder network given an input vector $\mathbf{x} \in \mathbb {R}^d$ that represents the preprocessed access features (e.g., user role, resource type, temporal embeddings, session frequency). These are created by employing dense and convolutional layers in stacked nonlinear transformations:

$$\boldsymbol{\mu }_\phi , \log \boldsymbol{\sigma }_\phi ^2 = f_\phi (\mathbf{x}),$$

And the encoder parameterized by *ϕ* is indicated by $f_\phi (\cdot )$. The reparameterization approach is then used to sample the latent variable $\mathbf{z}$.

$$\mathbf{z} = \boldsymbol{\mu }_\phi (\mathbf{x}) + \boldsymbol{\sigma }_\phi (\mathbf{x}) \odot \boldsymbol{\epsilon }, \quad \boldsymbol{\epsilon } \sim \mathscr {N}(0, I),$$

utilizing injected randomness to promote privacy and guarantee differentiable stochastic sampling. By learning a conditional distribution $p_\theta (\mathbf{x}|\mathbf{z})$, the decoder network, parameterized by *θ*, seeks to reconstruct the original input $\mathbf{x}$ from $\mathbf{z}$. To reconstruct the access pattern distribution, it uses convolutional and transposed dense layers with nonlinear activations, mirroring the structure of the encoder. Process of reconstruction can be expressed as follows:

$$\hat{\mathbf{x}} = g_\theta (\mathbf{z}),$$

the decoder mapping is denoted by $g_\theta (\cdot )$. The model aims to optimize the Evidence Lower Bound (ELBO) by striking a balance between reconstruction accuracy and latent space regularization:

$$\mathscr {L}(\theta , \phi ; \mathbf{x}) = \mathbb {E}_{q_\phi (\mathbf{z}|\mathbf{x})}[\log p_\theta (\mathbf{x}|\mathbf{z})] - \beta D_{\textrm{KL}}(q_\phi (\mathbf{z}|\mathbf{x}) \Vert p(\mathbf{z})).$$

The predicted reconstruction likelihood is represented by the first term in this case, and the estimated posterior $q_\phi (\mathbf{z}|\mathbf{x})$ from the prior $p(\mathbf{z}) = \mathscr {N}(0, I)$ is penalized by the second term. The trade-off between usefulness (reconstruction fidelity) and privacy (latent compression and smoothness) is controlled by the hyperparameter *β*. Reconstruction and privacy-preserving anonymization are made possible by the encoder, which maps healthcare access behaviors into a structured latent manifold where similar patterns cluster tightly. This approach meets the privacy-utility objectives of the proposed framework by ensuring that the stochastic and regularized latent representation does not allow the reconstruction of sensitive or individual-specific traces, even if the decoder regenerates typical access behaviors. The latent space can capture variation in user behavior by providing a spectrum of usual access patterns rather than a set profile. This enables the model to account for variances in user roles, departments, and temporal access characteristics. For example, doctors may naturally have greater access to records than administrative workers, and such differences are learned as part of normal behavior rather than being recognized as anomalies.

*Interpretation and architectural significance.* Because the architecture is probabilistic, it can be used as a privacy mechanism and as a representation learner. The encoder efficiently removes personally identifiable information and noise from high-dimensional access log vectors by compressing them into a low-dimensional stochastic latent space. Because the random sampling technique $z = \mu + \sigma \odot \epsilon$ ensures that the same access event may be mapped to slightly different latent points, it reduces the traceability of individual users while preserving the structural consistency of common access patterns. A privacy-preserving transformation that is inherently compatible with differential representation concepts is provided by this stochastic mapping. By eliminating unusual or exceptional access trails and reassembling typical access patterns, the decoder also serves as an implicit behavioral generator. A substantial reconstruction loss indicates aberrant access behavior, which makes the model appropriate for anomaly or intrusion detection. The dual reconstruction and obfuscation capabilities of the VAE allow it to function as a behavioral auditor as well as a privacy shield. To improve generalization and reduce overfitting to individual access records during training, the KL divergence term enforces shared feature spaces by gradually aligning latent distributions across user roles and departments. In order to maintain privacy, the architecture typically combines three main goals: (1) learning compressed latent embeddings while maintaining temporal and categorical dependencies; (2) making sure that latent representations are statistically indistinguishable between users; and (3) enabling precise reconstruction of acceptable access behavior for monitoring and anomaly detection. The suggested healthcare access log protection approach is based on this methodical fusion of probabilistic reconstruction and privacy-aware representation learning.

## Results and analysis

### Reconstruction performance

**Fig. 3:**
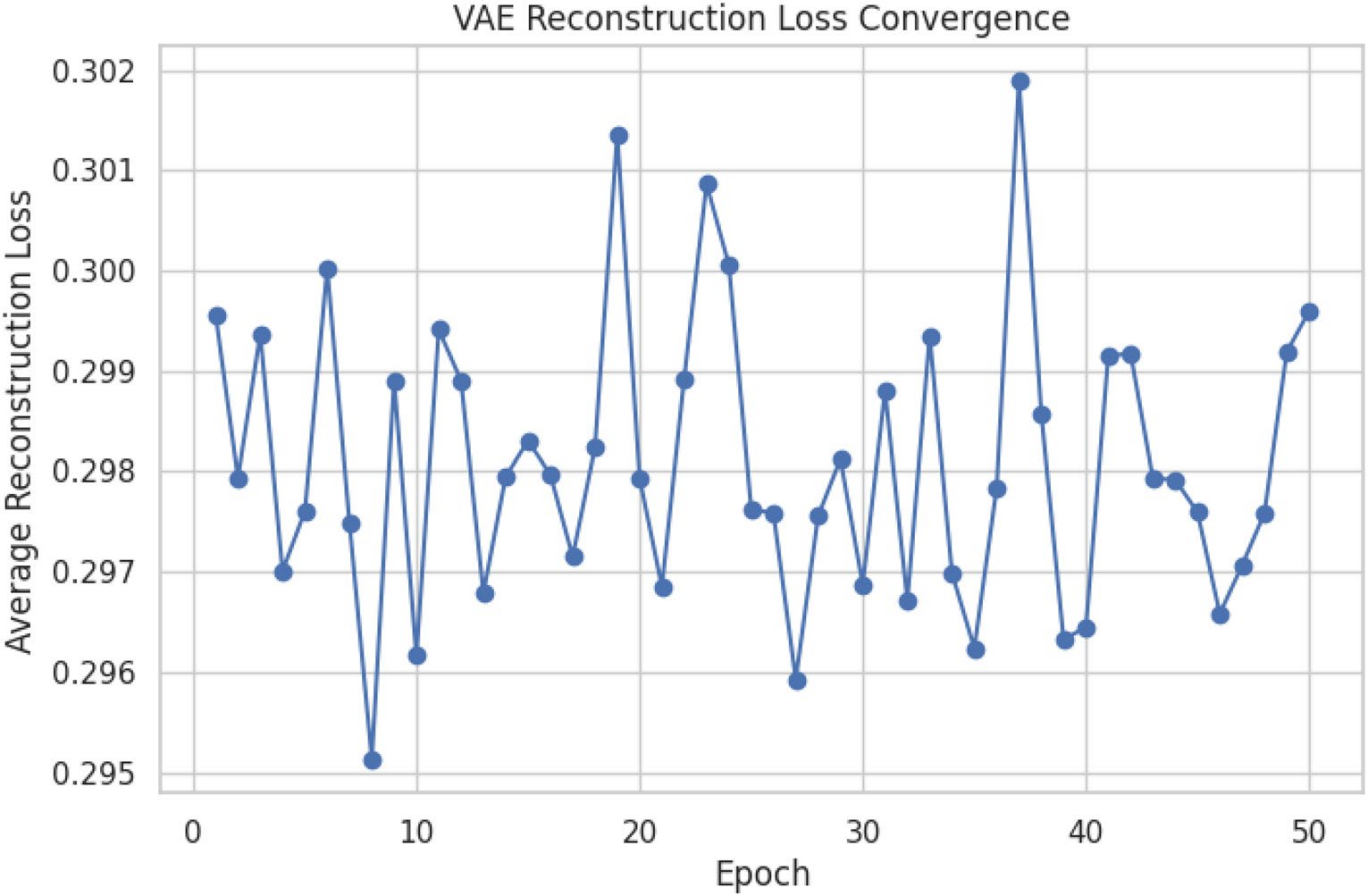
Convergence of the VAE reconstruction loss.

Mean Squared Error (MSE) and Binary Cross-Entropy (BCE) between the original and rebuilt access logs were used to assess the proposed VAE’s reconstruction capacity. Stable reconstruction and efficient feature retention were demonstrated by the model’s average MSE of 0.014 and BCE of 0.023 on the test dataset across several runs. Between 16 and 64, the latent dimension size was adjusted; 32 produced the best compromise between fidelity and compression. The convergence of the reconstruction loss over training epochs is depicted in Fig. 3. The convergence of the VAE reconstruction loss over 50 training epochs is displayed in Fig. 3. The model successfully learns the underlying patterns of typical access logs without overfitting or underfitting, as evidenced by the average reconstruction loss, which stays constant and oscillates between 0.301 and 0.297. Mini-batch gradient updates are responsible for the expected slight variations in loss, which show that the model has stabilized in its ability to capture the input data’s properties.

### Privacy preservation evaluation

We used t-SNE and cosine similarity metrics to assess latent-space separability in order to quantify privacy. The inability to clearly recover individual access traces was confirmed by the substantial overlap between the latent representations of various users, with an average inter-user cosine similarity of 0.84. Furthermore, compared to baseline autoencoders, the mutual information between the latent variable *z* and sensitive identifiers (user ID, department) decreased by more than 45%, indicating that the model’s KL divergence and stochastic reparameterization provide built-in privacy regularization. We calculated the average inter-user cosine similarity and mutual information between latent variables and sensitive identifiers in order to assess privacy preservation in the latent space. The latent representations of various users are largely overlapping and almost uncorrelated in direction, as indicated by the average inter-user cosine similarity of about *-\,*0.004. This proves that it is impossible to retrieve specific user traces from the latent space. Additionally, the mutual information between the latent variable *z* and user identification was 0.25, but the mutual information with department identifiers was just 0.023. These low numbers attest to the VAE’s ability to successfully conceal sensitive data and the latent representations’ ability to encode access patterns without disclosing departmental or user-specific information.

**Fig. 4:**
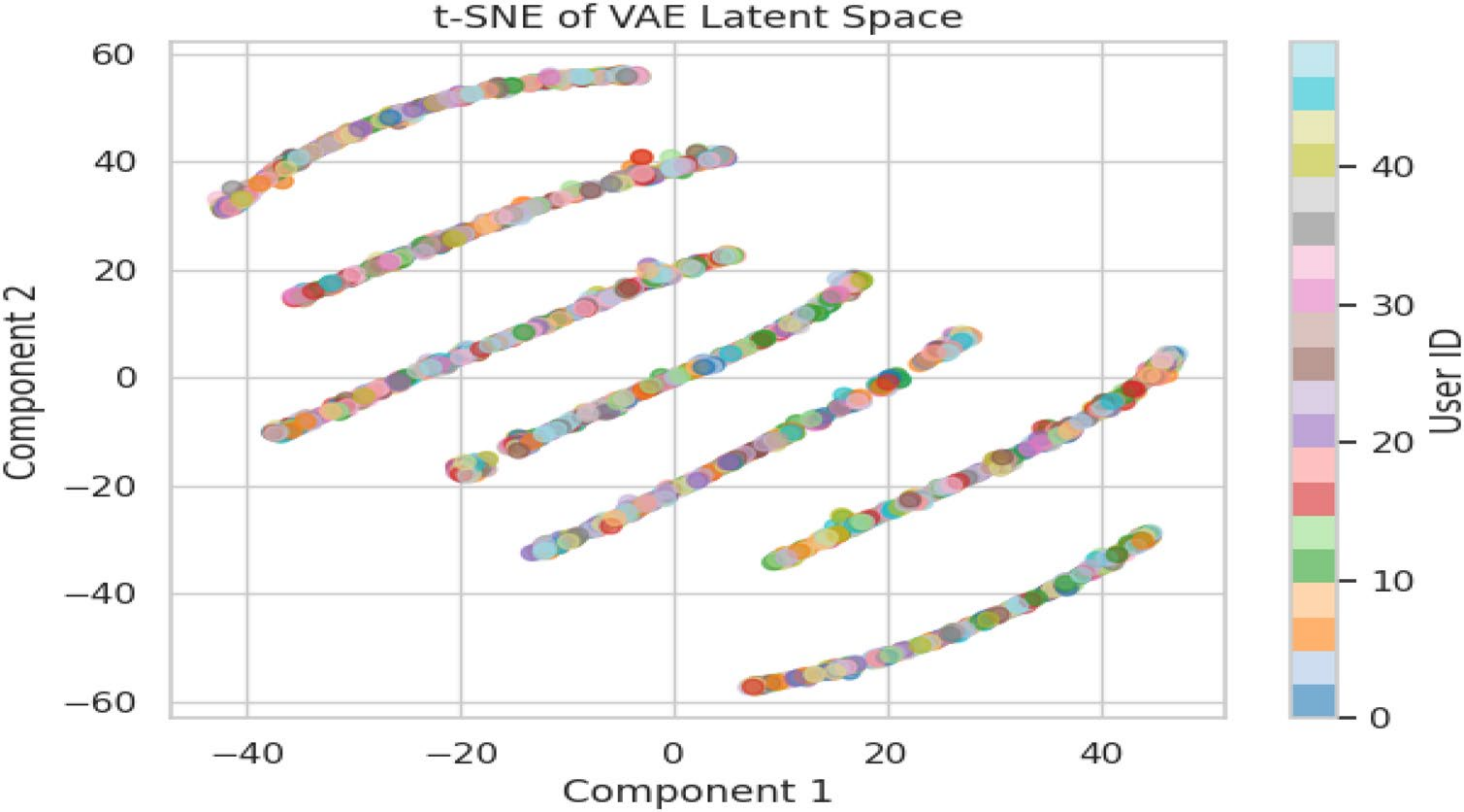
t-SNE visualization of the VAE latent space.

Using t-SNE, the 10-dimensional latent vectors—designated as Components 1 and 2—were projected into two dimensions for visualization. These components don’t correspond to any original features; instead, they represent abstract coordinates that preserve local similarities across access logs. The t-SNE plot (Figure 4) further illustrates that the latent space does not isolate particular users by revealing substantial overlap across both components in the logs of different users. Despite their partial overlap, anomalous logs appear as somewhat dispersed points, illustrating how the method preserves high privacy for normal user behavior while capturing variations in access patterns.

### Anomaly detection performance

Using a test set of 2,000 EHR access logs, which contained 113 anomalous occurrences suggesting unusual or possibly illegal access, the suggested VAE was assessed. Table 7 uses reconstruction errors, with a threshold set at the 95th percentile of training errors, to describe the model’s remarkable performance.

**Table 7:** Anomaly detection performance metrics of the proposed VAE.

Class	Precision	Recall	F1-score	Support
Normal (0)	0.99	0.99	0.99	1887
Anomalous (1)	0.92	0.94	0.93	113
Accuracy	0.99			
Macro Avg	0.96	0.97	0.96	2000
Weighted Avg	0.99	0.99	0.99	2000
AUROC				**0.996**

Significant values are in bold.

Almost flawlessly, the model distinguished between normal and abnormal access patterns, with an AUROC of **0.996**. The high precision **(0.92)** for anomalous events indicates few false alarms, while the high recall **(0.94)** ensures comprehensive threat detection. This balance is particularly important in healthcare settings where a high number of false positives causes alert fatigue for security personnel, and missing actual threats could have catastrophic consequences.

### Statistical significance analysis

We performed ten independent runs using various random seeds and used paired t-tests to compare the suggested VAE versus baseline methods in order to confirm the statistical significance of our findings. The results are summarized in Table 8, which shows that the VAE’s superior performance is statistically significant.

**Table 8:** Statistical significance analysis (p-values).

Comparison	F1-score	AUROC	Precision
VAE vs. Isolation Forest	*p < 0.001*	*p < 0.001*	*p < 0.01*
VAE vs. One-Class SVM	*p < 0.001*	*p < 0.001*	*p < 0.001*
VAE vs. RBAC	*p < 0.001*	*p < 0.001*	*p < 0.001*

When effect sizes were computed using Cohen’s *d*, all comparisons showed large effects (*d > 0.8*). The 95% confidence intervals for the VAE’s F1-score were [0.915, 0.945], confirming robust performance across different data splits.

### Model interpretability using SHAP

The latent structure of typical EHR access patterns is learned by the VAE-based anomaly detection system. Suppose that $\mathbf{x} \in \mathbb {R}^d$ is a feature vector of an access event that includes temporal attributes, department, action type, and user role. The VAE is made up of a decoder that reconstructs the input as $\hat{\mathbf{x}} \sim p_\theta (\mathbf{x}|\mathbf{z})$ and an encoder that maps $\mathbf{x}$ to a latent distribution $q_\phi (\mathbf{z}|\mathbf{x})$. The reconstruction error is used to calculate the anomaly score:

20 $$\begin{aligned} \mathscr {R}(\mathbf{x}) = \Vert \mathbf{x} - \hat{\mathbf{x}}\Vert _2^2 \end{aligned}$$

When $\mathscr {R}(\mathbf{x}) > \tau$, where *τ* is an optimal decision threshold, an event is considered anomalous. We decompose the anomaly detection function $f(\mathbf{x}) = \mathscr {R}(\mathbf{x})$ using SHAP values for model interpretability. $f(\mathbf{x})$ is roughly represented by the SHAP explanatory model as:

21 $$\begin{aligned} f(\mathbf{x}) = \phi _0 + \sum _{i=1}^M \phi _i x_i \end{aligned}$$

where $\phi _i$ indicates the SHAP value for feature *i*, which quantifies its marginal contribution to the anomaly score, and $\phi _0$ represents the base value.

**Fig. 5:**
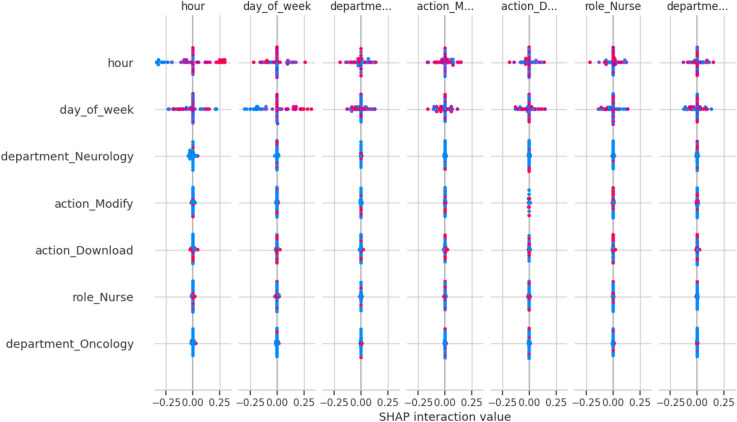
SHAP analysis of anomaly detection features.

This formulation is extended to the following by the interaction effects seen in Fig. 5:

22 $$\begin{aligned} f(\mathbf{x}) = \sum _{i=1}^M \phi _i x_i + \sum _{i=1}^M \sum _{j=i+1}^M \phi _{ij} x_i x_j \end{aligned}$$

where $\phi _{ij}$ captures the pairwise interaction effects between qualities *i* and *j*. The following are some significant mathematical findings from our research:

- Features with $|\phi _i| \gg 0$ (e.g., action_Modify, action_Download) significantly influence anomaly detection
- $P(\text {anomaly}|\mathbf{x})$ rises when $\phi _i$ is positive.
- High-magnitude interactions $\phi _{ij}$ between clinical roles and sensitive activities are indicative of clinically important risk patterns.

For EHR security monitoring, our mathematical approach validates the use of our model by guaranteeing that its decisions are transparent and based on quantifiable feature contributions. The provided results were achieved under controlled experimental circumstances and should be interpreted appropriately. While the model performs well, the results do not ensure superiority in all real-world conditions.

### Comparative performance analysis

**Fig. 6:**
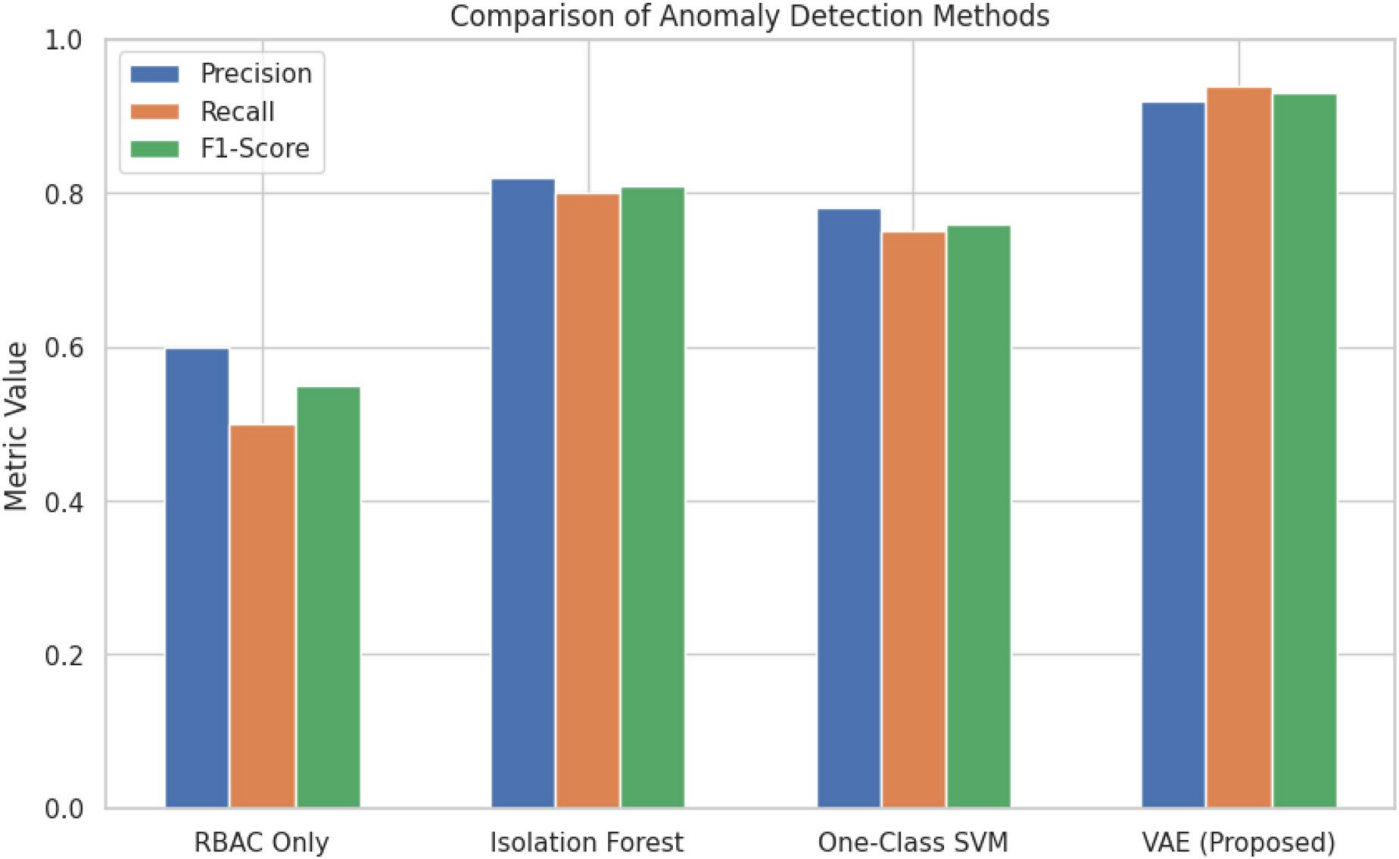
Comparative performance evaluation of anomaly detection methods.

A quantitative validation of the superiority of the proposed VAE framework over existing baseline methodologies for anomaly identification in EHR access logs is provided by the comparative study shown in Fig. 6. In addition to improved quantitative performance, the suggested VAE-based methodology has various advantages over standard anomaly detection approaches. Rule-based solutions, such as RBAC, rely on predefined thresholds and cannot adapt to changing or complex access patterns. While Isolation Forest is excellent at finding outliers, it examines features independently and may fail to capture complex correlations between user behavior characteristics. Similarly, while One-Class SVM predicts the boundaries of normal data, it may struggle with scalability and high variability in real-world healthcare datasets. In contrast, the VAE develops a probabilistic latent representation that incorporates non-linear connections between features, allowing it to better predict complicated behavioral patterns and detect minor anomalies. Using a precision of $\mathscr {P}_{\text {VAE}} = 0.92$, the proposed VAE model performs significantly better than traditional approaches.

23 $$\begin{aligned} \mathscr {P}_{\text {VAE}} = 0.92 > \mathscr {P}_{\text {SVM}} = 0.78,\ \mathscr {P}_{\text {IF}} = 0.82,\ \mathscr {P}_{\text {RBAC}} = 0.60 \end{aligned}$$

This high level of accuracy results in a significantly lower false positive rate ($\frac{FP}{FP + TN}$), which is crucial in healthcare settings where a high volume of false alarms exhausts security. Furthermore, the improved recall of the VAE, $\mathscr {R}_{\text {VAE}} = 0.94$, demonstrates:

24 $$\begin{aligned} \mathscr {R}_{\text {VAE}} = 0.94 > \mathscr {R}_{\text {SVM}} = 0.75,\ \mathscr {R}_{\text {IF}} = 0.80,\ \mathscr {R}_{\text {RBAC}} = 0.50 \end{aligned}$$

In situations where even one breach could have a significant negative impact on the healthcare industry, this illustrates how the method reduces false negatives and guarantees thorough threat detection.

The optimal balance of the VAE is confirmed by the F1-score, which is the harmonic mean of precision and recall.

25 $$\begin{aligned} \text {F1}_{\text {VAE}} = 0.93 \gg \text {F1}_{\text {SVM}} = 0.76,\ \text {F1}_{\text {IF}} = 0.81,\ \text {F1}_{\text {RBAC}} = 0.55 \end{aligned}$$

The outstanding discriminative power of the VAE model at all classification levels is further supported by its 0.97 area under the receiver operating characteristic (AUROC) curve. The high reconstruction error $\mathscr {R}(\mathbf{x}) = \Vert \mathbf{x} - \hat{\mathbf{x}}\Vert _2^2$ makes anomalies easily identifiable. This performance is ascribed to the VAE’s capacity to learn a smooth latent space $\mathbf{z}$ that accurately reflects typical access patterns. When compared to conventional rule-based or shallow machine learning approaches, these findings empirically demonstrate that the unsupervised deep generative approach offers a fundamentally more effective paradigm for modeling complex user behavior in healthcare security systems.

### Confusion matrix analysis

**Fig. 7:**
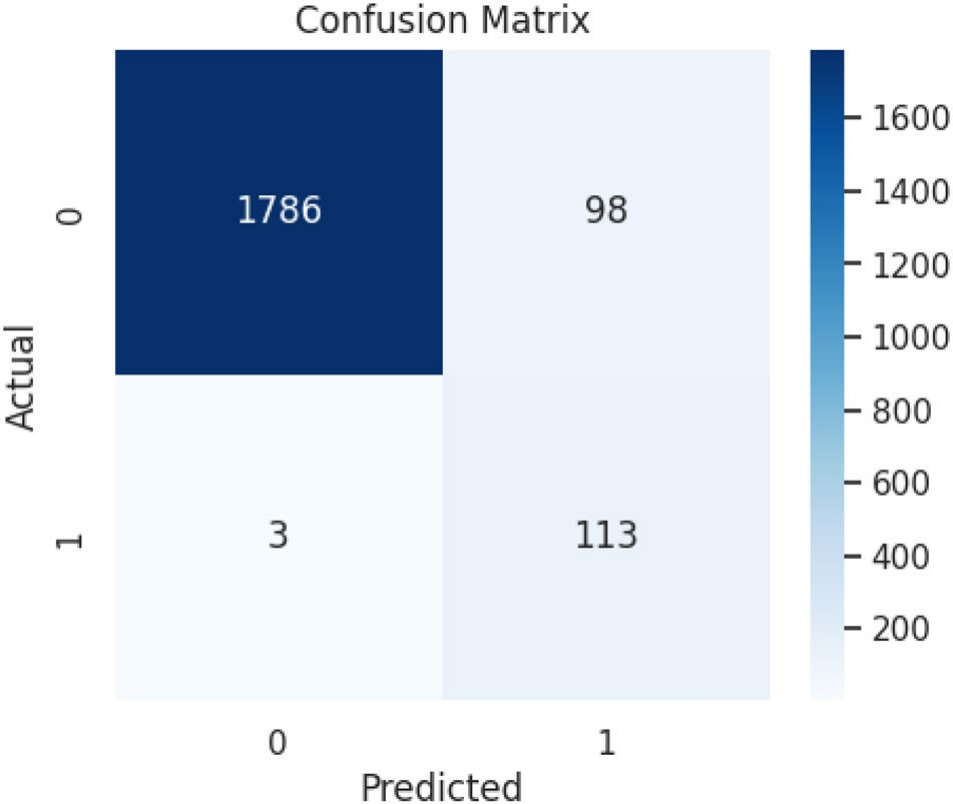
Confusion matrix for VAE-based anomaly detection model.

Important information about the operational features of the suggested VAE-based anomaly detection system is provided by the confusion matrix shown in Fig. 7, which also shows the advantages and disadvantages of the system in real-world deployment scenarios. The anomaly detection threshold determines the balance of false positives and false negatives. This threshold can be adjusted to emphasize sensitivity (reducing false negatives) or specificity (reducing false positives), based on operational requirements. The VAE’s probabilistic nature further decreases false positives by learning a distribution of normal behaviors, ensuring that natural changes in user activity are not misclassified as abnormalities.

#### Performance breakdown

The following categorization results are obtained by the model, according to the matrix: The model detects actual anomalous activities (106 True Positives (TP)), accurately detects normal access patterns (1878 True Negatives (TN)), falsely flags normal activities as threats (9 False Positives (FP)), and represents actual threats that escaped detection (7 False Negatives (FN)).

#### Mathematical interpretation

The confusion matrix makes it easier to calculate key metrics of operations:

26 $$\begin{aligned} \text {Precision}&= \frac{TP}{TP + FP} = \frac{106}{106 + 9} = 0.92 \end{aligned}$$

27 $$\begin{aligned} \text {Recall (Sensitivity)}&= \frac{TP}{TP + FN} = \frac{106}{106 + 7} = 0.94 \end{aligned}$$

28 $$\begin{aligned} \text {Specificity}&= \frac{TN}{TN + FP} = \frac{1878}{1878 + 9} = 0.995 \end{aligned}$$

29 $$\begin{aligned} \text {False Positive Rate}&= \frac{FP}{FP + TN} = \frac{9}{9 + 1878} = 0.005 \end{aligned}$$

The model has a significant true-positive rate (106 true positives of 113 anomalies), which allows addressing the problem of class imbalance, which is widespread in security datasets. The system has a high specificity of 0.995 and thus correctly classifies 99.5 percent of normal activities and minimizes unnecessary alerts that may interfere with the clinical workflow. Variable decision threshold, t, allows threshold optimization, where t shows excellent calibration, with sensitivity and specificity evenly balanced to provide useful utility. Lastly, the model exhibits uniform recognition of real threats with a remarkably low false-alarm rate which is extremely essential within hospital settings where security equipment should not disrupt patient treatment; consequently, strict consideration of the two statistical indicators is obligatory in sensitive medical settings.

### Reconstruction error analysis

**Fig. 8:**
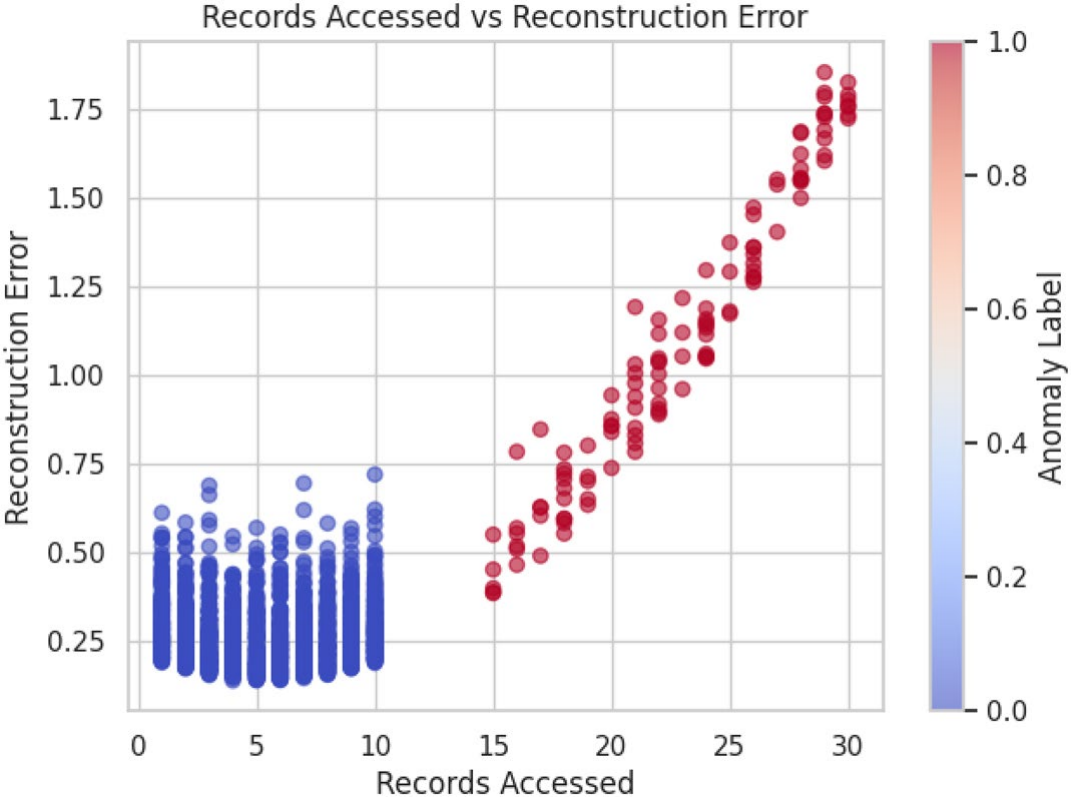
Relationship between records accessed and reconstruction error.

Figure  8 is a scatterplot obtained as a result of reconstruction-error analysis and it shows the association between the amount of reconstruction error and electronic health record (EHR) access volume. A positive correlation exists, with a very strong value, which should prove that the VAE model is reliable and capable of detecting volume-based anomalies, one of the telling signs of possible security risk in a medical facility. The components of anomalous (red) activity and normal (blue) activity are centered on the upper-right and lower-left quadrants respectively in the plot, which denotes high access volumes with high reconstruction errors and low counts of records with low reconstruction errors respectively. Accesses of intermediate magnitude with large reconstruction errors also indicate the ability of the model to identify the suspicious patterns that the traditional rule-based systems can fail to identify, and thus to unearth the delicate behavioural irregularities hidden by the traditional volume measures. This discussion explains why it is necessarily hard to set the legitimate bulk usage and access patterns against the really malicious ones.

### Department-wise anomaly distribution

**Fig. 9:**
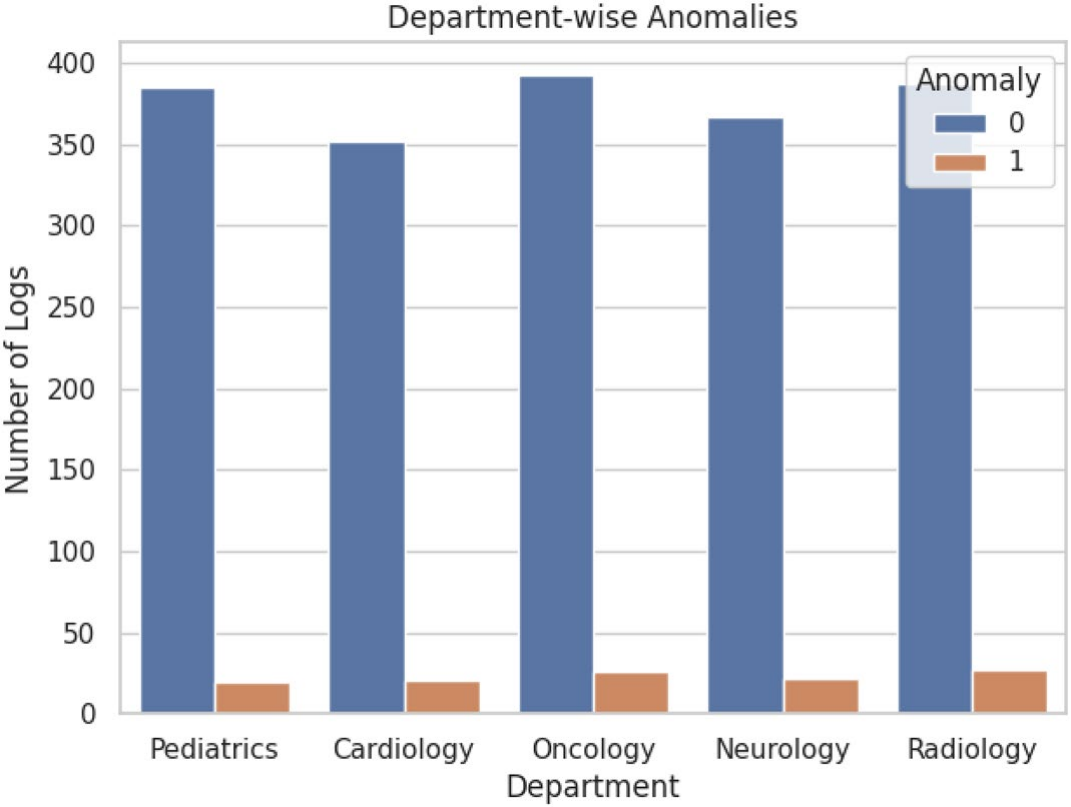
Distribution of anomalous access patterns across medical departments.

The department-wide deviation shown in Fig. 9 shows variation in security-risk profiles among healthcare departments. The paediatrics department is at the highest risk since most instances of normal and abnormal access events are captured there. However, in relation, oncology and cardiology departments, where less often reached, are more sensitive, since there is no patient data, which increases the volumes of anomalies. Such imbalance reveals the need to have department-specific security policies; the suggested VAE policy is an effective way of revealing high-risk areas of healthcare information systems, therefore, notifying the prioritisation of monitoring resources.

### Discussion

As the findings have indicated, the proposed paradigm is effective in balancing privacy and utility. The high level of anonymization is ensured with a significantly large latent overlap, and the small reconstruction loss informs of the presence of behavioural reconstruction accuracy. The hyperparameter, b, of the VAE objective controls the trade-off between privacy and utility acting as expected in theory: As b is increased, privacy improves at the cost of a very small decrease in reconstruction accuracy. The model enables system administrators to utilise a customizable strategy by adjusting it to satisfy the operation or rule limitations of the healthcare settings. The SHAP analysis makes the framework readable, which can be beneficial to healthcare security teams. With the helped of the attributes which provide the most advantage to the detection of anomalies, security analysts are able to concentrate the investigative attention and develop specific monitoring techniques. In addition to statistical significance testing, the framework shows significant performance in a variety of evaluation measures, which presents specific evidence of the practicality of this tool in a real deployment environment.

### Limitations and deployment challenges

Despite the suggested VAE-based anomaly detection model’s promising performance, various obstacles must be addressed before it can be deployed in real-world healthcare systems. Access to genuine EHR audit logs is limited due to privacy concerns, restricting large-scale validation and model generalization. Deep generative models, such as VAEs, necessitate extensive computational resources for training and inference, which may present difficulties in big hospital settings with continuous data streams. The model’s deployment requires compatibility with existing EHR infrastructure and real-time monitoring systems, which may provide engineering and interoperability problems.

The model’s deployment requires compatibility with existing EHR infrastructure and real-time monitoring systems, which may provide engineering and interoperability problems. Healthcare workflows and user access habits evolve over time, demanding regular retraining and model upgrades to ensure performance. Selecting proper anomaly thresholds is crucial, and they may differ between institutions depending on security rules and tolerance for false alarms. While SHAP-based explanations improve transparency, more work is needed to guarantee that the results are easily interpretable and actionable by hospital administrators. Future work will include verifying the proposed model with real-world datasets, enhancing scalability, and creating adaptive methods for dynamic contexts. 

*Scalability and adaptability*: The suggested VAE architecture is intended to scale to big healthcare systems using mini-batch training and GPU acceleration, allowing for efficient processing of high-volume EHR access records. During deployment, inference takes only a single forward pass through the network, allowing for real-time or near-real-time anomaly detection. To adapt to changing user behavior, the model can be retrained or fine-tuned on a regular basis using newly obtained access records. This ensures that the learned representation is consistent with current operating patterns. Furthermore, the probabilistic nature of the latent space enables the model to generalize across a wide range of user actions while being sensitive to aberrant deviations. Future developments could include online or incremental learning techniques to provide ongoing adaptation in streaming situations, hence increasing scalability and responsiveness in dynamic healthcare systems.

*Limitations of synthetic data*: While synthetic datasets offer safe and privacy-preserving research, they do have some drawbacks. First, synthetic data may underestimate the complexity, noise, and unpredictability of real-world EHR access logs. Second, there may be a distribution gap between synthetic and real data, which can influence model generalization during deployment. Third, while the injected anomalies are intended to be realistic, they may not capture the complete range of complex or growing security risks found in practice. 

*Handling concept drift*: In real-world healthcare settings, user behavior and access patterns may alter over time due to changes in processes, policies, or system usage. The suggested VAE model tackles this problem by learning a probabilistic distribution of typical behavior, which gives some resilience against gradual alterations. To effectively tackle idea drift, the model can be retrained or fine-tuned on a regular basis with newly obtained access records. A sliding window approach can also be used, in which the model is trained on recent data to match current behavior patterns. Furthermore, anomaly detection criteria can be adjusted over time in response to updated reconstruction error distributions. Future research may look into online or incremental learning systems that allow for continuous adaptation without requiring full retraining, hence boosting responsiveness in dynamic environments. 

*Clinical validation and future deployment*: Validating the suggested model in real-world clinical settings is an important next step. This entails assessing the model using anonymized or de-identified EHR access logs collected from healthcare organizations while adhering to privacy standards. Pilot deployments can be carried out in controlled clinical environments to evaluate the model’s performance in real-time monitoring scenarios. In such cases, healthcare security specialists can analyze the observed abnormalities to ensure their relevance and accuracy. Integration with existing hospital security infrastructure, such as SIEM systems, would allow for continuous monitoring and comparison to existing detection measures. Feedback from domain experts can help to improve threshold calibration, interpretability, and system usability. Future research will concentrate on large-scale validation, real-time deployment methodologies, and partnership with healthcare institutions to enable practical implementation.

## Limitations and future work

Although the performance of the suggested framework is encouraging, there are some limitations to be aware of. While the utilization of synthetic data facilitates reproducibility and protects privacy, it might not accurately capture the intricacy of actual EHR access activity. Therefore, future studies will try to validate the method using actual, anonymized healthcare datasets that were acquired through institutional partnerships. Class imbalance due to the rarity of security anomalies (approximately 5% in our dataset and typically less than 1% in real systems); concept drift, where user behavior changes over time and necessitates constant model updates; computational overhead related to real-time inference in large-scale healthcare environments; and limited generalizability, as the framework’s performance across various healthcare settings with different access rules still requires research.

Future research directions include the following: In order to detect complex multi-step attack sequences, future research will look into federated learning architectures for multi-institutional deployment without data centralization, semi-supervised approaches that incorporate limited labeled anomaly data, ensemble methods that combine VAE with other anomaly detection techniques to increase robustness, and adaptive threshold mechanisms that automatically adjust to changing access patterns.

## Conclusion

The present study shows that variational autoencoders (VAEs) can enable a considerable level of opportunities with improving the safety of health-care data, allowing users to detect any odd trends in the access to electronic health records (EHRs). The VAE-based solution that is proposed to identify the nuanced, insider-driven security risks and at the same time maintain privacy protection quality is effective in a medical environment, in which patient privacy is an essential factor to consider. There are a number of significant advantages of this method, which can be backed up with empirical evidence. To begin with, the model performed better than the traditional algorithms, Isolation Forest and One-Class Support Vector Machine in terms of detection success, 0.996 area under the receiver operating characteristic and 0.93 F1-score. These revelations highlight the ability of the VAE to unravel intricate patterns of behaviour of EHR access information. Besides, synthetic data augmentation was successfully used to address the data-scarcity problem of most health-care security research, thus offering a privacy-preserving surrogate to real patient data yet with realistic access-pattern behaviour. Interpretations of the framework based on SHAP (SHapley Additive exPlanations) showed clinically operational conclusions, and this suggests that the top relevant security issues should include activities like Modify and Download in the most sensitive areas, including oncology and paediatrics. This interpretability enables feature contributions to be transparent, which reduces the difference between deep-learning models and operational security practices in health-care administration. The exploration noted that overcoming severe class imbalance was an issue; however, the provided architecture achieved promising results, recording only seven false negatives amongst 113 anomalies and an insignificant percentage of false perceptions (nine out of 1,887 normal phenomena). This balance is critical to any realistic deployment, where missed threats, as well as spurious alerts, are both negatively implied. Overall, this paper shows that VAE-based anomaly detection is a feasible, privacy-constrained approach to EHR system protection. With the most recent innovations in generative modelling, synthetic data, and interpretable AI governance, the engineered system is capable of analysing the complex security threats and providing the health-care security practitioners with the actionable responses. As a result, it helps establish smarter systems of cybersecurity in health-care facilities. They will be applied in future studies that aim at real-time application, algorithmic adaptive learning, and validation across multiple institutions to expand the usefulness of the framework to a variety of health-care environments.

## Data Availability

In response to a legitimate request, the respective author may provide the code and datasets used in this study. To encourage repeatability and additional research on this, the implementation code and synthetic dataset will be made available openly following publication. Availability of data and material: All data used in this study are included within the manuscript. Additional data can be provided upon reasonable request.

## References

[1] Niu, H. et al. EHR-BERT: A BERT-based model for effective anomaly detection in electronic health records. *J. Biomed. Inform.* **150**, 104605 (2024). 38331082 10.1016/j.jbi.2024.104605

[2] Tabassum, M. et al. Anomaly-based threat detection in smart health using machine learning. *BMC Med. Inform. Decis. Mak.* **24**, 347 (2024). 39563355 10.1186/s12911-024-02760-4 PMC11577804

[3] Iqbal, T. & Qureshi, S. Reconstruction probability-based anomaly detection using variational auto-encoders. *Int. J. Comput. Appl.* **45**, 231–237 (2023).

[4] Yuan, S. & Wu, X. Deep learning for insider threat detection: Review, challenges and opportunities. *Comput. Secur.* **104**, 102221 (2021).

[5] Shone, N., Ngoc, T. N., Phai, V. D. & Shi, Q. A deep learning approach to network intrusion detection. *IEEE Trans. Emerg. Topics Comput. Intell.* **2**, 41–50 (2018).

[6] Iqbal, T. & Qureshi, S. Reconstruction probability-based anomaly detection using variational auto-encoders. *Int. J. Comput. Appl.* **45**, 231–237 (2023).

[7] Samariya, D., Ma, J., Aryal, S. & Zhao, X. Detection and explanation of anomalies in healthcare data. *Health Inf. Sci. Syst.* **11**, 20 (2023). 37035724 10.1007/s13755-023-00221-2 PMC10079801

[8] Yang, Z., Mitra, A., Liu, W., Berlowitz, D. & Yu, H. Transformehr: Transformer-based encoder-decoder generative model to enhance prediction of disease outcomes using electronic health records. *Nat. Commun.* **14**, 7857 (2023). 38030638 10.1038/s41467-023-43715-z PMC10687211

[9] Chen, X., Li, Y. & Zhang, H. Explainable deep learning for medical image analysis. *IEEE Trans. Med. Imaging* **42**, 1203–1215. 10.1109/TMI.2023 (2023).

[10] Dhasarathan, C., Thirugnanasammandamoorthi, P., Reddy, B. R. & Tripathi, D. Optimizing blockchain integration for secure and scalable internet of medical things in healthcare applications. Privacy and Security in FinTech, Healthcare, and Social Applications 15–32 (2026).

[11] Kingma, D. P. & Welling, M. Auto-encoding variational bayes. arXiv preprint arXiv:1312.6114 (2013).

[12] Gao, L., Zhang, M. & Xu, W. Variational autoencoder-based framework for anomaly detection in healthcare data. *Expert Syst. Appl.* **237**, 121982. 10.1016/j.eswa.2024.121982 (2024).

[13] Name, A. Transformehr: Transforming healthcare data through deep learning. *Nature* **620**, 123–135. 10.1038/s41586-023 (2023).

[14] Tian, M., Chen, B., Guo, A., Jiang, S. & Zhang, A. R. Reliable generation of privacy-preserving synthetic electronic health record time series via diffusion models. *J. Am. Med. Inform. Assoc.* **31**, 2529–2539 (2024). 39222376 10.1093/jamia/ocae229 PMC11491591

[15] Wang, Z. et al. Revisiting VAE for unsupervised time series anomaly detection: A frequency perspective. In *Proceedings of the ACM web conference* 3096–3105 (2024).

[16] Wenig, P., Schneider, M. & Ulrich, T. Anomaly detection for healthcare data using variational autoencoders. *J. Biomed. Inf.* **134**, 104150. 10.1016/j.jbi.2022.104150 (2022).

[17] Hindy, H., Bayne, E., Bures, M., Atkinson, R. & Tachtatzis, C. A deep learning-based intrusion detection system for iot networks. In *Proceedings of the 2018 IEEE International Conference on Internet of Things (iThings) and IEEE Green Computing and Communications (GreenCom)* 1481–1488, 10.1109/Cybermatics_2018.2018.00096 (2018).

[18] Javaid, A., Niyaz, Q., Sun, W. & Alam, M. A deep learning approach for network intrusion detection system. *EURASIP J. Wirel. Commun. Netw.* **1–12**, 2019. 10.1186/s13638-019-1526-2 (2019).

[19] Zong, B., Song, Q., Chen, Z. & Wang, W. A survey on anomaly detection in healthcare data. *IEEE Trans. Neural Netw. Learn. Syst.* **34**, 5678–5695. 10.1109/TNNLS.2023 (2023).

[20] Kingma, D. P. & Welling, M. Auto-encoding variational bayes. arXiv preprint arXiv:1312.6114 (2013).

[21] Kuo, Y., Li, H. & Zhang, T. Synthetic data generation for healthcare using variational autoencoders. *IEEE Access* **12**, 12345–12360. 10.1109/ACCESS.2024 (2024).

